# Biopsy‐free screening for glioma

**DOI:** 10.15252/emmm.201809484

**Published:** 2018-11-06

**Authors:** Alexandre Pellan Cheng, Philip Burnham, Iwijn De Vlaminck

**Affiliations:** ^1^ Meinig School of Biomedical Engineering Cornell University Ithaca NY USA

**Keywords:** Biomarkers & Diagnostic Imaging, Cancer, Chromatin, Epigenetics, Genomics & Functional Genomics

## Abstract

Circulating tumor DNA (ctDNA) is a promising diagnostic marker for many cancers and can be noninvasively assayed from blood. For diagnosing glioma, this approach has unfortunately proven to be of limited use since glioma contribute minimal ctDNA to the blood circulation. A more promising avenue may therefore be to hunt for ctDNA in cerebrospinal fluid (CSF). The study by Mouliere *et al* in this issue of *EMBO Molecular Medicine* demonstrates that shallow whole‐genome sequencing of CSF‐cfDNA can be used to detect copy number alterations in glioma‐derived ctDNA, providing a low cost strategy to screen for glioma.

Glioma is one of the most common types of brain tumors. Each year, more than 200,000 individuals are diagnosed with glioma worldwide (Parkin *et al*, 2005). The outlook for these patients remains abysmal: in the United States, more than half of glioma patients die from their disease. Initial diagnosis of glioma is usually performed by neuroimaging, and more refined disease classification requires tumor resection or biopsy. There is therefore an urgent need for informative molecular markers to diagnose, classify and monitor glioma (Westphal & Lamszus, 2015).

For many human cancers, molecular diagnostic tests that assay cell‐free DNA (cfDNA) in the blood circulation have shown exciting promise (Bettegowda *et al*, 2014). These molecules of circulating DNA are derived from the genomes of dying cells from across the body, including fast proliferating cells within tumors, and provide a noninvasive, biopsy‐free window into the genetic makeup of tumors. For glioma, this approach has unfortunately proven to be of limited use: only a small subset of high‐grade gliomas give rise to detectable levels of tumor DNA in plasma (Bettegowda *et al*, 2014). This is likely because gliomas hide behind the blood–brain barrier; few molecules of circulating tumor DNA (ctDNA) make it across this barrier and into the blood.

A more promising avenue may therefore be to hunt for ctDNA in cerebrospinal fluid (CSF). The presence of tumor DNA in CSF was first reported in 1995 by Harker Rhodes *et al*, who used allele specific PCR to detect tumor‐derived p53 DNA in the CSF of a glioblastoma patient (Harker Rhodes *et al*, 1995). In 2015, several groups reported the use of high‐throughput sequencing technologies to characterize ctDNA in CSF. Pan *et al* (2015) used both droplet digital PCR and targeted amplicon sequencing to screen for brain tumor mutations in CSF of patients with primary and metastatic brain tumors. Wang *et al* (2015) reported detectable levels of ctDNA in 74% of cases of primary central nervous system (CNS) malignancies of diverse histology and location within the CNS. De Mattos‐Arruda *et al* (2015) compared the abundance of ctDNA in plasma and CSF and were able to directly establish that ctDNA is much more prevalent in CSF for patients with disease restricted to the CNS. These studies however relied on assays that require *a priori* knowledge of the cancer's genetic makeup, expensive deep sequencing (Wang *et al*, 2015), or a combination thereof, and are consequently of limited use in disease screening applications.

In this issue of *EMBO Molecular Medicine*, Mouliere *et al* (2018) report a low‐cost strategy to screen for glioma. The authors’ approach takes advantage of shallow whole‐genome sequencing (sWGS) of CSF‐cfDNA and careful examination of structural features on both short and long molecular length scales (Fig 1).

**Figure 1:**
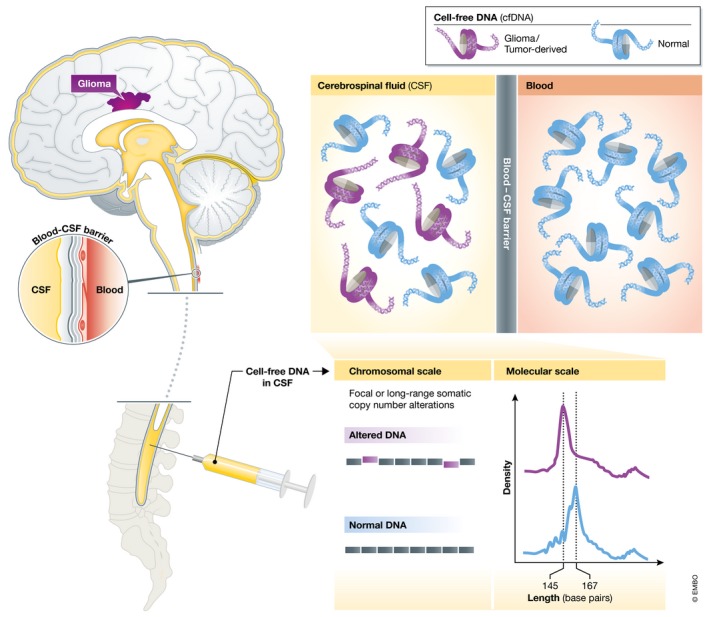
Study overview and schematic of low‐cost strategy to screen for glioma cfDNA derived from glioma is much more prevalent in CSF than in plasma. In this issue of *EMBO Molecular Medicine*, Mouliere *et al* report a low‐cost strategy to screen for glioma that takes advantage of shallow whole‐genome sequencing of CSF‐cfDNA and careful examination of structural features on both short and long molecular length scales.

On the length scale of genes and chromosomes, the authors looked for somatic copy number alterations (SCNAs) in CSF‐sWGS data. SCNAs were detected in the CSF of five out of 13 glioma patients as focal events confined to individual genes and as large events spanning entire chromosomes. Similar SCNAs were not seen identified in matched plasma samples, confirming earlier reports that cfDNA in CSF better represents the genomic alterations of brain tumors than cfDNA in plasma (Bettegowda *et al*, 2014; De Mattos‐Arruda *et al*, 2015). For a single patient, SCNAs detected in CSF recapitulated the combined alterations present in multiple solid‐tumor subparts. This is an exciting result because it indicates that CSF‐cfDNA samples the tumor mass globally, and is able to identify alterations that may be missed by biopsies that sample locally.

To study structural features in CSF‐cfDNA on molecular scales, the authors implemented paired‐end DNA sequencing, which provides sequence information from both ends of the molecules, and obtained the size distribution of cfDNA fragments in CSF. This analysis revealed a shortening of fragments in the CSF of patients with primary brain tumors. A similar shortening has been observed for ctDNA in plasma (Underhill *et al*, 2016). In addition, the authors report the curious observation that the amplitude of periodic 10 bp oscillations in the fragment size distribution is negatively correlated with the levels of SCNAs. This novel signature may provide yet another way to detect the presence of tumor‐derived cfDNA in CSF that does not require knowledge of point mutations or SCNAs within the tumor.

Cell‐free DNA in CSF provides a compelling, information‐rich window into the genetic makeup and biology of brain cancers (Harker Rhodes *et al,* 1995; Wang *et al,* 2015, Pentsova *et al,* 2016). The study by Mouliere *et al* is an important early step in the creation of diagnostic protocols that take advantage of low‐cost screening via CSF‐cfDNA to identify samples where more expensive, confirmatory testing is needed. First however, further study is necessary to validate the concepts put forward in this paper in a larger cohort of patients.

## Conflict of interest

The authors declare that they have no conflict of interest.
